# Response of *Staphylococcus aureus* to combination of virulent bacteriophage vB_SauM-515A1 and linezolid

**DOI:** 10.3389/fmicb.2024.1519312

**Published:** 2024-12-20

**Authors:** Narina K. Abdraimova, Egor A. Shitikov, Dmitry A. Bespiatykh, Roman B. Gorodnichev, Ksenia M. Klimina, Vladimir A. Veselovsky, Daria I. Boldyreva, Alexandra S. Bogdanova, Dmitry V. Klinov, Maria A. Kornienko

**Affiliations:** ^1^Lopukhin Federal Research and Clinical Center of Physical-Chemical Medicine of Federal Medical Biological Agency, Moscow, Russia; ^2^Moscow Institute of Physics and Technology, National Research University, Dolgoprudny, Russia

**Keywords:** bacteriophage, *Staphylococcus aureus*, linezolid, combination therapy, biofilm

## Abstract

The combined use of lytic bacteriophages with antibiotics is currently being explored as a strategy to enhance the effectiveness of infectious disease therapies, including those caused by *Staphylococcus aureus*. In this study, we investigated the synergistic potential of bacteriophage vB_SauM-515A1 (*Herelleviridae* family) and the first-line antibiotic linezolid against the methicillin-resistant *S. aureus* strain SA0413Rev. A checkerboard assay revealed a significant synergistic effect against planktonic cells (FIC = 0.225): a combination of 1/8 MIC of linezolid and 0.01 MOI of the bacteriophage completely inhibited bacterial growth. However, the impact on biofilm-associated cells depended on the treatment sequence. Sequential administration resulted in antagonism, while simultaneous application demonstrated a synergistic effect, as confirmed through scanning electron microscopy. Transcriptomic analysis of *S. aureus* SA0413Rev under the combined influence of linezolid (1/4 MIC) and bacteriophage vB_SauM-515A1 (10 MOI) predominantly reflected changes associated with productive bacteriophage infection, including alterations in nucleotide metabolism, activation of prophage regions, and virulence factors. Furthermore, both agents affected energy and carbon metabolism. These findings contribute to the development of combination therapy approaches for infections caused by *S. aureus* and highlight the importance of optimizing treatment conditions for maximal therapeutic efficacy.

## 1 Introduction

*Staphylococcus aureus* is one of the most significant pathogens in clinical practice, responsible for a wide range of infections in humans, including skin infections, pneumonia, endocarditis, and sepsis (Cheung et al., 2021; Linz et al., 2023; Naghavi et al., 2024). The growing prevalence of drug-resistant strains, particularly methicillin-resistant *S. aureus* (MRSA), has significantly complicated treatment strategies and is linked to increased mortality rates (Guo et al., 2020).

In light of rising antibiotic resistance, increasing attention is being directed toward the study of bacteriophages (phages)—natural bacterial predators. The most promising group of phages for treating staphylococcal infections is the *Herelleviridae* family, whose members exhibit strictly lytic life cycles and broad host ranges. The efficacy of these phages has been demonstrated in various studies, including *in vitro* and *in vivo* experiments, as well as clinical cases (Petrovic Fabijan et al., 2020; Plumet et al., 2022; Liu et al., 2024).

Beyond the individual use of phages, there is growing interest in the combined application of phages and antibiotics (Li et al., 2021a; Łusiak-Szelachowska et al., 2022). Such studies aim to identify optimal combinations and dosages that enhance treatment effectiveness while ensuring safety of application. For instance, the inclusion of phages in therapeutic regimens may reduce the required doses of antibiotics, thereby reducing the risk of side effects. Additionally, combining agents could reduce the likelihood of bacterial resistance development (Łusiak-Szelachowska et al., 2022).

The combined application of antibacterial agents can result in various outcomes, such as additive effects, synergy, or antagonism. In the case of additive effects, the combined action of two agents equals the sum of their individual effects. When the combined efficacy exceeds this additive effect, synergy is achieved. However, when one component limits the other’s activity, resulting in reduced overall antibacterial effectiveness, this is considered antagonism (Gu Liu et al., 2020).

In practical applications, it is essential to explore combinations of phages with current antibacterial drugs effective against MRSA. Vancomycin, a glycopeptide antibiotic, is a key first-line treatment for MRSA. However, due to the emergence of resistance (Cong et al., 2020), alternative treatments are now used, including linezolid, a synthetic oxazolidinone. Linezolid inhibits protein biosynthesis by binding irreversibly to the 30S and 50S ribosomal subunits, thereby disrupting the formation of the 70S initiation complex and inhibiting peptide chain elongation (Hashemian et al., 2018).

It should be noted that prolonged linezolid use can lead to serious side effects, such as lactic acidosis (Palenzuela et al., 2005; Sharma et al., 2020). Furthermore, the use of linezolid against microorganisms with minimum inhibitory concentrations (MICs) of 4 μg/mL (at which *S. aureus* is classified as sensitive; bacterium is considered resistant when MIC ≥ 8, according to the Clinical and Laboratory Standards Institute (CLSI) standards) or higher may lead to suboptimal clinical outcomes, potentially due to issues with drug delivery and plasma concentration variability (Hui et al., 2022; Azzouz and Preuss, 2024).

The combined use of lytic phages with linezolid, including at sub-inhibitory concentrations, has been proposed as a strategy to improve therapeutic outcomes and mitigate adverse effects. Previous studies have demonstrated a synergistic effect of linezolid and phages on planktonic and biofilm-associated bacterial cells (Wang et al., 2020; Li et al., 2021b). However, other researchers have raised concerns about combining bacteriostatic antibiotics like linezolid with phages, as the replication of phages might be hindered (Berryhill et al., 2021). These discrepancies have also been observed in animal model experiments (Chhibber et al., 2013; Albac et al., 2020; Prazak et al., 2021).

In this study, we investigated the individual and combined effects of sub-inhibitory concentrations of phage vB_SauM-515A1, a member of the *Herelleviridae* family, and linezolid on *S. aureus*. The combined action of these two agents was evaluated against both planktonic cells and biofilms. To further characterize the interaction between the phage and linezolid, transcriptional analysis of the infected bacterial strain was performed. This study aims to expand our understanding of the mechanisms underlying the combined use of lytic phages and antibiotics against *S. aureus*, which is critical for optimizing therapeutic efficacy.

## 2 Materials and methods

### 2.1 Bacterial strain and bacteriophage

The previously characterized *S. aureus* SA0413Rev strain was selected from the strain collection at the Lopukhin Federal Research and Clinical Center of Physical-Chemical Medicine (Kornienko et al., 2020b). Bacterial cultures were grown in lysogeny broth (LB) (Oxoid, UK) or on LB agar (Oxoid) at 37°C. Susceptibility to linezolid (Sigma-Aldrich, USA) was determined according to the CLSI standards. Multilocus sequence typing (MLST) of the strain was performed following the standard method (Enright et al., 2000).

The lytic phage vB_SauM-515A1, used in this study, belongs to the *Kayvirus* genus within the *Herelleviridae* family and exhibits typical myovirus morphology. It was originally isolated from a commercial *Staphylococcus* bacteriophage cocktail (batch P332) produced by Microgen (Russia), utilizing the *S. aureus* strain SA515 (spa-type t008, ST8) as the host. This phage has been extensively characterized in previous studies (Kornienko et al., 2020b; Kuptsov et al., 2022).

For all subsequent experiments, the phage was propagated as follows: an overnight culture of *S. aureus* SA0413Rev (OD_620_ = 0.6) was diluted 1:100 in fresh LB broth and grown to an OD_620_ = 0.12. The phage was then added at a multiplicity of infection (MOI) of 0.1, and the culture was incubated at 37°C with shaking (200 rpm) for 24 h. The resulting phage lysate was centrifuged at 10,000 *g* for 10 min at 4°C to remove bacterial debris. The supernatant was subsequently filtered through a 0.22 μm membrane filter (Merck Millipore, USA) to obtain a sterile phage preparation.

### 2.2 Evaluation of phage-antibiotic synergy in planktonic bacterial cultures

The individual and combined effects of the phage and linezolid were investigated using a checkerboard assay as previously described (Loganathan et al., 2024), with some modifications. Briefly, phage vB_SauM-515A1 was added to the wells of a 96-well flat-bottom polystyrene microplate (Thermo Scientific, USA) containing LB, with final concentrations ranging from 0 to 10^5^ PFU/mL (serial 1:10 dilutions) in the horizontal wells. Linezolid was added vertically, with final concentrations ranging from 0 to 32 μg/mL (serial 1:2 dilutions). Bacterial suspensions (OD_620_ = 0.12; 1.2 × 10^8^ CFU/mL) were added to achieve a final concentration of 10^4^ CFU/mL (Colony-forming unit). Positive controls consisted of LB medium inoculated with the bacterial strain, while sterile LB was used for negative controls. The final volume in each well was 200 μL. The phage and antibiotic effects were monitored by measuring optical density at 620 nm every hour for 24 h at 37°C using a Microplate Reader Flex-A (Allsheng, China). All experiments were performed in triplicate. The results were converted to percentage reduction relative to the positive control using the following formula (Gu Liu et al., 2020):


$$\text{Reduction}\%=\frac{\text{GrowthControl}-\text{Treatment}}{\text{GrowthControl}}\times 100$$

To assess the combination effect, the fractional inhibitory concentration (FIC) index was calculated according to the following formula (Jo et al., 2016):


FIC=FIC_antibiotic+FIC_phage=

$$\frac{\text{C\_antibiotic}}{\text{MIC\_antibiotic}}+\frac{\text{C\_phage}}{\text{MIC\_phage}}$$

where MIC_antibiotic and MIC_phage represent the MICs of the antibiotic and phage, and C_antibiotic and C_phage are the respective inhibitory concentrations of the antibiotic and phage in combinations. FIC values were interpreted as follows: synergy (FIC < 0.5), additive effect (0.5 ≤ FIC < 2), and antagonism (FIC ≥ 2).

### 2.3 Biofilm formation assay

The biofilm formation assay followed the previously described method (Cassat et al., 2007) with modifications. Bacterial suspensions in the exponential growth phase (OD_620_ = 0.12) were inoculated into wells containing tryptic soy broth with 1% glucose (TSBg, Himedia, India) at a final concentration of 10^4^ cells per well, and incubated for 48 h at 37°C without shaking. After incubation, the wells were gently washed with sterile phosphate-buffered saline (PBS) to remove planktonic cells and stained with 0.1% crystal violet (Sigma-Aldrich, USA). Sterile medium was used as the negative control. Biofilm formation was quantified by measuring the optical density at 570 nm. All experiments were performed in triplicate.

Biofilm formation was classified according to following criteria (Stepanović et al., 2007): no biofilm (OD ≤ OD_*C*_), weak biofilm (OD_*C*_ < OD ≤ 2 × OD_*C*_), moderate biofilm (2 × OD_*C*_ < OD ≤ 4 × OD_*C*_), and strong biofilm (OD > 4 × OD_*C*_), where OD_*C*_ = average OD of the negative control + (3 × SD of the negative control).

### 2.4 Evaluation of antibacterial treatments on biofilm associated cells

The evaluation of antimicrobial treatments on biofilm-associated cells was performed in accordance with the study by Dickey and Perrot (2019), with minor modifications. Biofilms were grown for 48 h and washed with sterile PBS as aforementioned. After washing, 200 μL of TSBg containing various combinations of antibacterial agents were added to each well of a 96-well microplate. Bacteriophage (1 MOI) or linezolid at two concentrations (2 or 10 MIC) was used for individual treatment. For simultaneous treatments, both bacteriophage and linezolid were applied at the corresponding concentrations. The negative control contained TSBg without any antibacterial agents. Incubation was conducted for 48 h at 37°C. For sequential treatment, 200 μL of TSBg with bacteriophage (1 MOI) was added and incubated for 24 h at 37°C. Subsequently, linezolid (2 or 10 MIC) was added in an amount not exceeding 5% of the total volume, followed by an additional 24 h incubation at 37°C. After treatment, biofilms were disrupted by pipetting, and viable cells were determined by serial dilution plating on LB agar, followed by incubation at 37°C for 24 h. All experiments were performed in triplicate.

The interaction effects of the phage and antibiotic were calculated using the coefficient of interaction (COI) as described by Kumaran et al. (2018):


$$\text{COI}={\text{logAP}}_{\text{R}}-\left({\text{logA}}_{\text{R}}+{\text{logP}}_{\text{R}}\right)$$

where A_*R*_ - reduction in bacterial counts by treatment with antibiotic, P_*R*_ - reduction in bacterial counts by treatment with phage, and AP_*R*_ - reduction by the combined treatment (staggered or simultaneous). Results were interpreted as synergistic (COI > 0), additive (COI = 0), or antagonistic (COI < 0). A mixed model analysis was performed to assess the significance of COI results, with a *P*-value < 0.05 considered significant.

### 2.5 Biofilm scanning electron microscopy (SEM) imaging

Biofilms were grown directly on catheters (Apexmed International B.V., Netherlands) placed in a 24-well plate (Corning, USA). Exponentially growing bacterial suspensions (OD_620_ = 0.12) were added to the wells containing TSBg medium at a final concentration of 10^4^ cells per well. After 5 days of incubation at 37°C, the medium was replaced with fresh TSBg. Antibacterial treatment was carried out as described above in Section 2.4, using bacteriophage at 108 PFU/mL and linezolid at 2 MIC. The samples were then processed for scanning electron microscopy (SEM) imaging as previously described (Sokolova et al., 2019). Briefly, biofilms were fixed with 2.5% glutaraldehyde (Sigma-Aldrich, USA) in PBS for 2 h at room temperature, and then washed with PBS three times. The dehydration was performed using the following series of ethanol–water mixtures: 10, 20, 30, 40, 50, 60, 70, 70, 80, 90, 96, and 96% (12 steps, 5 min each). Next, the samples were chemically dried with a 10 min incubation in HMDS: ethanol 1:1 mixture, two 10 min incubations in 100% HMDS, and one incubation in 100% HMDS until complete evaporation. Preliminary coated with a ∼10 nm gold-palladium alloy layer using Eiko IB 3 ion coater (Japan), the samples were observed with a Zeiss Merlin microscope equipped with Gemini II Electron Optics (Zeiss, Oberkochen, Germany). The images were acquired at low accelerating voltage (2 kV) and low probe current (100 pA) via HE-SE2 detector. The FIJI software (NIH, Bethesda, MD, USA) was used to process SEM images (Schindelin et al., 2012).

### 2.6 One-step growth curve of bacteriophage

The one-step growth curve of phage vB_SauM-515A1 on the *S. aureus* strain SA0413Rev was performed as previously described (Kornienko et al., 2020b), with the addition of a sub-inhibitory concentration of linezolid (1/4 MIC). Briefly, *S. aureus* SA0413Rev cells in the early exponential phase (OD_620_ = 0.12) were infected with the phage (MOI 0.001) in the presence of linezolid. The mixture was incubated for 7 min at 37°C, followed by centrifugation for 4 min at 10,000*g*. The pellet was resuspended in 300 μl of LB. Aliquots of 10 μl were collected at 15, 20, 30, 40, 50, 60, 70, 80, 90, 100, and 120 min post-infection. Samples were treated with 1% chloroform, and the number of phage particles was determined using the double agar overlay assay (Kropinski et al., 2009). A sample without the addition of the antibiotic served as a positive control. All experiments were performed in triplicate.

### 2.7 DNA extraction

Genomic DNA was isolated from an overnight culture of *S. aureus* SA0413Rev (OD_620_ = 0.6) grown in LB broth using the Wizard Genomic DNA Purification Kit (Promega, USA) following the manufacturer’s protocol. DNA concentration and quality were assessed using a Qubit 4 Fluorometer and Nanodrop ND-1000 (Thermo Fisher Scientific).

### 2.8 Illumina library preparation and sequencing

A total of 100 ng of isolated DNA was used for library preparation with the KAPA HyperPlus Kit (Roche, Switzerland), following the manufacturer’s protocol. The library was subjected to final cleanup using KAPA HyperPure Beads (Roche, Switzerland). Library size distribution and quality were assessed using a High Sensitivity DNA chip (Agilent Technologies), and quantification was performed using the Quant-iT DNA Assay Kit, High Sensitivity (Thermo Fisher Scientific).

The DNA libraries were sequenced on the HiSeq 2500 platform (Illumina, USA) according to the manufacturer’s instructions. The following reagent kits were used: HiSeq Rapid PE Cluster Kit v2, HiSeq Rapid SBS Kit v2 (200 cycles), and HiSeq Rapid PE FlowCell v2, along with a 2% PhiX spike-in control.

### 2.9 Oxford nanopore library preparation and sequencing

Libraries were prepared according to the manufacturer’s protocol using NEB reagents, with long reads generated on the PromethION platform (Oxford Nanopore Technologies, UK). The sequencing libraries were prepared with the ligation sequencing kit SQK-LSK109 and the native barcoding expansion kit EXP-NBD196, and run on an R9.4.1 (FLO-PRO002) flow cell. Basecalling was performed using Guppy v6.5.7 with default parameters (high accuracy model, minimum quality score ≥ 7).

### 2.10 Total RNA extraction

For transcriptomic analysis, *S. aureus* cultures (OD_620_ = 0.12) were treated with either the phage, linezolid, or a combination of both. The antibiotic was used at a concentration of 1/4 MIC, while the phage was added at an MOI of 10. Untreated cultures served as controls. At each time point (5, 20, 30, and 50 min after exposure to the antibacterial agents), 1 mL of the culture was collected, centrifuged (3 min at 5,000 *g*) at 4°C, and the cell pellets were immediately frozen at −70°C. All experiments were performed in triplicate.

RNA was extracted from the frozen cell pellets using the MagMAX mirVana Total RNA Isolation Kit (Thermo Fisher Scientific, Lithuania) on the KingFisher Flex Purification System (Thermo Fisher Scientific, USA) following the manufacturer’s protocol. RNA was treated with DNase using the Turbo DNA-Free Kit (Thermo Fisher Scientific) in a 50 μl volume and further purified using Agencourt RNAClean XP beads (Beckman Coulter, USA). The total RNA concentration was measured with the Quant-iT Ribogreen RNA Assay Kit (Thermo Fisher Scientific), and the quality of the extracted RNA was verified using an Agilent Bioanalyzer with RNA 6000 Pico Chips (Agilent Technologies, USA).

### 2.11 RNA library preparation and sequencing

For transcriptomic library preparation, 150 ng of total RNA was used as input. Ribosomal RNA was selectively removed using the Ribo-Zero Plus rRNA Depletion Kit (Illumina, USA), followed by library preparation with the KAPA RNA Hyper Kit (Roche, Switzerland) according to the manufacturer’s protocol. RNA purification steps involved the use of RNA Clean XP magnetic beads (Beckman Coulter, Brea, USA), and final library cleanup was done with Agencourt AMPure XP beads (Beckman Coulter, Brea, USA). Library size distribution and quality were assessed using an Agilent High Sensitivity DNA kit (Agilent Technologies, USA), and library concentration was determined using the Quant-iT DNA Assay Kit, High Sensitivity (Thermo Fisher Scientific, USA).

Equimolar amounts of all libraries (12 pM) were sequenced on the Illumina HiSeq platform using 2 × 100 bp paired-end reads with a 5% PhiX spike-in control. RNA-seq read data were deposited in the NCBI Sequence Read Archive under bioproject PRJNA1172966.

### 2.12 Bioinformatics analysis

#### 2.12.1 Genome assembly

Taxonomic confirmation of the sequenced reads was accomplished with Kraken2 v2.1.2 (Wood et al., 2019) and Bracken v2.8 (Lu et al., 2017). The quality assessment of short paired-end reads was performed using falco v1.2.1 (de Sena Brandine and Smith, 2019) and MultiQC v1.17 (Ewels et al., 2016). Adapters removal and reads filtering was performed using fastp v0.23.4 (Chen et al., 2018). Long-reads quality was evaluated with Nanoq v0.10.0 (Steinig and Coin, 2022). Prior to assembly, long reads were filtered with Filtlong v0.2.1^1^. Hybrid assembly was created using Unicycler v0.5.0 (Wick et al., 2017), Medaka v. 1.11.3^2^, MaSuRCA v. 4.1.0 (Zimin et al., 2013), and Polypolish v. 0.6.0 (Wick and Holt, 2022). PGAP 2023-10-03.build7061 (Tatusova et al., 2016) was used for annotating the assembly. Minimap2 v2.26-r1175 (Li, 2018) was used to map long reads to the assembly. BWA MEM v0.7.17-r1188 (Li and Durbin, 2009) was employed for mapping short reads to the assembly. Subsequently, SAMtools v1.17 (Danecek et al., 2021) and mosdepth v0.3.5 (Pedersen and Quinlan, 2018) were used to compile mapping statistics. Prophage sequences were identified using the PHASTEST web server (Wishart et al., 2023). Antibiotic resistance genes and resistance-conferring mutations were detected with ResFinder v.4.1 (Bortolaia et al., 2020). Virulence Finder 2.0 was employed to search for virulence genes, with an identity threshold of 90% and a minimum protein length of 60%^3^. The genome of the *S. aureus* SA0413Rev has been deposited in GenBank under accession number CP173176.

#### 2.12.2 Differential gene expression analysis

Sequenced reads were aligned to the reference *S. aureus* SA0413Rev genome (CP173176) using STAR v2.7.11a (Dobin et al., 2013). SAMtools v1.17 software was utilized for sorting and converting SAM files to BAM format, followed by indexing and subsequent statistical analysis. Mapping quality and coverage along genes were evaluated with QualiMap v2.2.2 (Okonechnikov et al., 2016), and individual reports were merged using MultiQC v1.17. Reads were assigned to genes using featureCounts v2.0.6 (Liao et al., 2014). Differential gene expression analysis utilized the edgeR v3.42.4 (Robinson et al., 2010) package for R. Genes with a false discovery rate (FDR) cutoff of 0.05 and a fold change (log_2_FC) threshold of | 1| (i.e., ≥| 2| -fold change) were deemed differentially expressed. For gene ontology (GO) enrichment analysis, GO categories were annotated using the PANNZER2 tool (Törönen et al., 2018) with a Positive Predictive Value (PPV) cutoff of 0.5. Gene ontology (GO) enrichment analysis was conducted using GOpiscator v1.0.5^4^.

#### 2.12.3 Statistical analysis and data visualization

Figures were created using R v4.3.0 (R Core Team, 2023) using the following packages: ggplot2 v3.5.1 (Wickham, 2016), cowplot v1.1.1 (Wilke, 2024), ggnewscale v0.5.0.9000^5^, ggh4x v0.2.8^6^, colorspace v2.1-0 (Zeileis et al., 2020), scales v1.3.0 (Wickham et al., 2023), gridtext v0.5.1^7^, and ComplexHeatmap v2.16.0 (Gu et al., 2016). For statistical analyses R packages rstatix v0.7.2 (Kassambara, 2023), DescTools v0.99.50 (Signorell, 2024), and Python 3.11 module statsmodels v 0.13.5^8^ were used.

## 3 Results

### 3.1 Overview of experimental strategy

To compare the effects of individual and combined antimicrobial agents, we used the phage vB_SauM-515A1 and linezolid, a key antibiotic against MRSA. The characteristics of *S. aureus* strain SA0413Rev and phage vB_SauM-515A1 have been described previously (Kornienko et al., 2020b). This strain was sensitive to both the phage and linezolid, belonged to the clinically relevant sequence type ST8, and exhibited spa type t008, as well as multidrug resistance. According to the biofilm formation assay, SA0413Rev was classified as a moderate biofilm producer.

To assess the effects of individual and combined antimicrobial agents on planktonic cells of *S. aureus* SA0413Rev, we employed the checkerboard assay, measuring cell growth dynamics over 24 h. The nature of the combined effect was evaluated using the fractional inhibitory concentration (FIC) index.

Since biofilm formation is a critical factor in the pathogenesis of *S. aureus* infections, we also investigated the ability of the strain to form biofilms and evaluated the impact of individual and combined antibacterial agents on biofilm-associated cells. We tested different treatment regimens, including the simultaneous administration of both agents and sequential treatment (phage followed by antibiotic). The results were interpreted using the coefficient of interaction (COI). Additionally, scanning electron microscopy was employed to visualize structural changes in the biofilms.

Based on one-step growth curves, we selected time points for the final stage of the study—evaluating the bacterial transcriptional response to the combined treatment. The complete genome of the SA0413Rev strain was sequenced and characterized (Supplementary Text).

### 3.2 Effect of individual and combined treatment with phage and linezolid on planktonic cells of *S. aureus*

**FIGURE 1:**
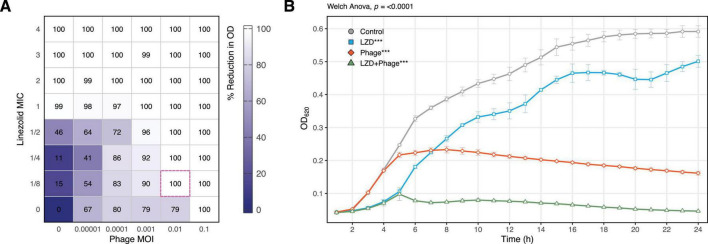
Synergistic effect of sublethal concentrations of vB_SauM-515A1 and linezolid against planktonic *S. aureus* SA0413Rev cells. **(A)** Checkerboard assay, the pink dashed outline indicates the greatest reduction at minimal concentration; **(B)** growth curves of the strain treated with 0.01 MOI phage, 1/8 MIC linezolid, and untreated control; LZD, linezolid. Statistical analysis was performed using Welch one-way ANOVA with Dunnett’s *post hoc*. test in comparison to control.

The evaluation of individual antimicrobial agents revealed that the minimum MOI for the phage and the MIC for linezolid that completely inhibited bacterial growth were 0.1 and 4 μg/mL, respectively (Figure 1). Lower concentrations of the antimicrobial agents slowed bacterial growth compared to the control but did not achieve complete inhibition.

Under combined treatment with sub-inhibitory concentrations (MOI 0.001 and 0.01 for the phage, and 1/8 to 1/2 MIC for linezolid), significant inhibition of bacterial growth was observed, with more than a 90% reduction in culture growth (Figure 1A). The FIC index for the combination was 0.225, indicating a synergistic effect. For instance, the combination of 1/8 MIC of linezolid and 0.01 MOI of the phage completely suppressed bacterial growth (Figure 1B).

### 3.3 Effect of phage and linezolid on *S. aureus* biofilms

**FIGURE 2:**
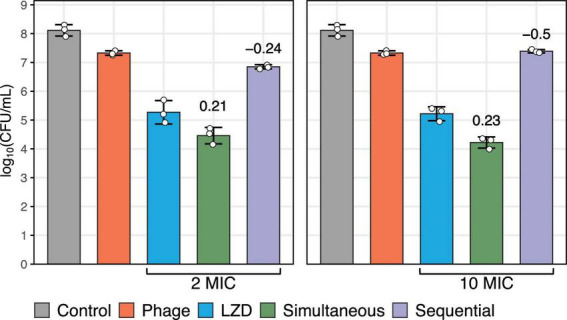
Biofilm-associated *S. aureus* SA0413Rev cells after 48 h of treatment with antimicrobial agents. The graph represents the number of viable bacteria. The control without antimicrobial agents is shown in gray. The individual effects of phage vB_SauM-515A1 (MOI 1) and linezolid (at 2 MIC and 10 MIC) are shown in red and blue, respectively. Simultaneous and sequential treatments with the antimicrobial agents are represented in green and violet, respectively. The values above the bars represent the calculated coefficient of interaction (COI).

The effect of antibacterial agents on biofilm-associated cells was evaluated based on CFU counts, which demonstrated a synergistic interaction when phage vB_SauM-515A1 was applied simultaneously with linezolid at concentrations of 2 MIC and 10 MIC (*p* < 0.05; COI > 0) (Figure 2). The individual application of linezolid at both concentrations showed moderate efficacy in reducing the number of viable cells, whereas the phage exhibited even lower activity under the same conditions. Sequential treatment of pre-formed biofilms with phage vB_SauM-515A1 followed by linezolid, regardless of the concentration, resulted in an antagonistic effect (*p* < 0.05; COI < 0). Notably, complete eradication of bacteria was not observed with any treatment method, while the most effective combination achieved only ∼4-log reduction.

**FIGURE 3:**
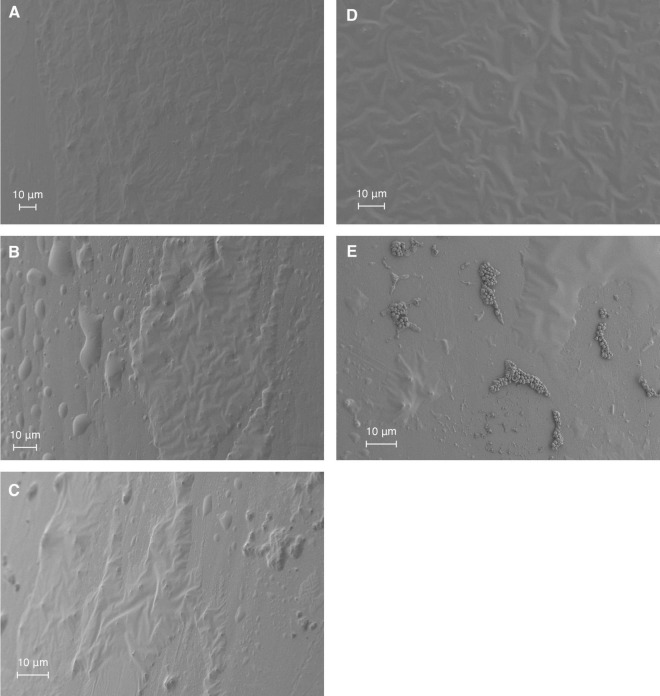
SEM visualization of *S. aureus* SA0413Rev biofilm on a catheter under antimicrobial treatment conditions. Biofilms were treated for 48 h with 2 MIC of linezolid **(A)**, phage vB_SauM-515A1 at MOI 1 **(B)**, or their combination: sequential treatment **(C)** or simultaneous application **(E)**. Untreated control biofilms are shown in panel **(D)**.

SEM was employed to visualize the structural effects of the antimicrobial agents and their combination (phage MOI 1 and linezolid concentration 2 MIC) on *S. aureus* biofilm formed on a catheter over 5 days (Figure 3). The extended growth period was chosen to mimic clinical scenarios, where biofilms typically form on medical devices over longer periods. As result, neither individual treatment with linezolid (Figure 3A) or phage (Figure 3B), nor their sequential application (phage followed by linezolid; Figure 3C), led to a noticeable reduction in the biofilm compared to the untreated control (Figure 3D). In contrast, simultaneous application of both agents resulted in a visible reduction of the biofilm matrix, with individual bacterial cells appearing in the field of view (Figure 3E).

### 3.4 Transcriptional response of *S. aureus* to individual and combined exposure to phage vB_SauM-515A1 and linezolid

The transcriptional response of *S. aureus* to antimicrobial agents was examined at 5, 20, 30, and 50-min time points, selected based on the growth curves of the phage’s lytic cycle (Supplementary Figure 1). Compared to the uninfected control, the combined treatment of strain SA0413Rev with the phage and antibiotic resulted in 839 differentially expressed genes (DEGs) (Supplementary Table 1). For cultures treated individually with linezolid or phage, the number of DEGs was 651 and 680, respectively.

**FIGURE 4:**
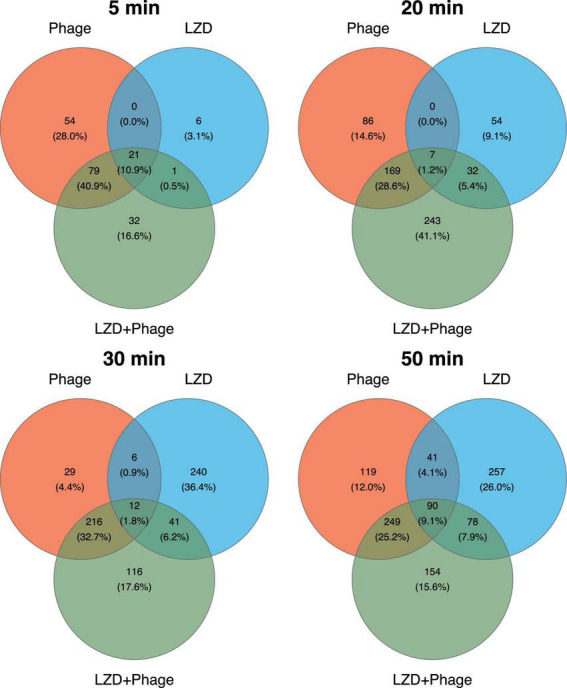
Venn diagrams representing the differentially expressed genes (DEGs) in *S. aureus* SA0413Rev under antimicrobial treatment across four time points. The numbers indicate the unique and overlapping DEGs between treatments (phage, linezolid (LZD), and their combination). Percentages in parentheses represent the proportion of DEGs identified at the corresponding time point.

Analysis of DEG distribution by agent and time point revealed that the highest number of DEGs was detected in the later stages of the experiment for any type of treatment (Figure 4). It was also found that, in the combined treatment case, the phage contributed the most to the transcriptional response. Common DEGs across all time points suggested a non-specific cellular response to both the individual and combined treatments.

**FIGURE 5:**
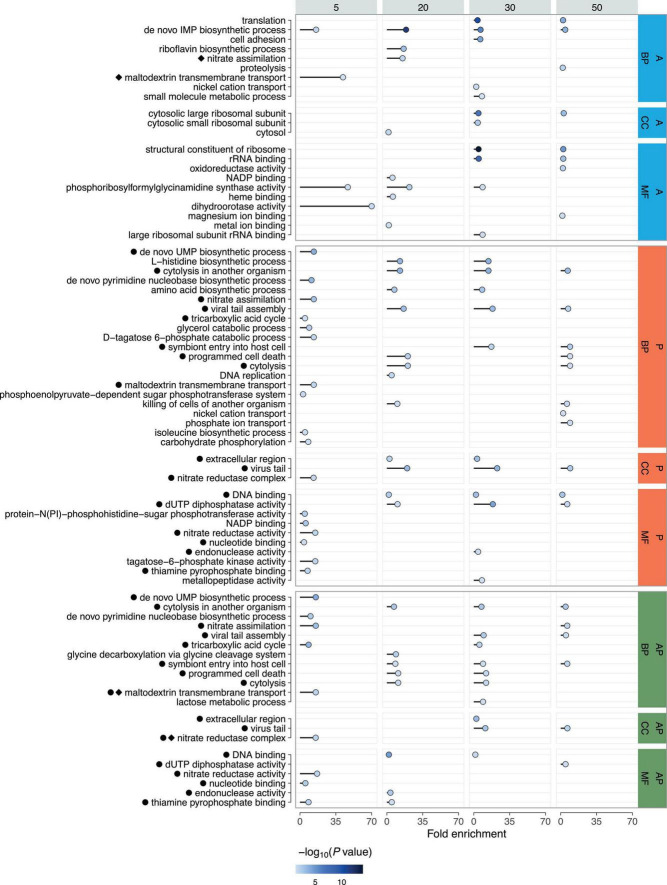
Gene Ontology (GO) analysis of DEGs across time points following phage (P), linezolid (A), and combined treatments (AP). Enrichment is shown for biological processes (BP), molecular functions (MF), and cellular components (CC). Fold Enrichment - the percentage of genes in the list belonging to a pathway, divided by the corresponding percentage in the background. The color intensity represents the *P*-value (-log10), with darker shades indicating stronger statistical significance. Co-occurring enriched GO terms for individual and combined treatments are indicated with black circles for phage and diamonds for antibiotic.

Functional analysis of the DEGs using gene ontology (GO) identified enrichment across all major categories: biological processes (BP), molecular functions (MF), and cellular components (CC) (Figure 5) (Supplementary Table 5). A more detailed examination of the BP category showed that transcriptional changes in enriched GO groups were mostly unidirectional across all time points and within each category (Supplementary Tables 1, 2).

Phage treatment at the 5-min time point resulted in decreased transcription of genes involved in nitrate assimilation (GO:0042128), glycerol catabolic process (GO:0019563), maltodextrin transmembrane transport (GO:0042956), D-tagatose 6-phosphate catabolic process (GO:2001059), phosphoenolpyruvate-dependent sugar phosphotransferase system (GO:0009401), tricarboxylic acid cycle (GO:0006099), isoleucine biosynthetic process (GO:0009097), and carbohydrate phosphorylation (GO:0046835). Upregulated genes were mainly associated with pyrimidine biosynthesis (GO:0044205; GO:0006207). At 20 and 30 min, all GO categories included only upregulated genes, representing processes such as amino acid metabolism (GO:0000105; GO:0008652), cytolysis in another organism (GO:0051715), prophage region activation (GO:0098003; GO:0006260), programmed cell death (GO:0012501), and cytolysis (GO:0019835; GO:0031640). At 30 min, amino acid metabolism (GO:0000105; GO:0008652), cytolysis in another organism (GO:0051715), and prophage region activation (GO:0098003; GO:0046718) were again detected, with similar categories observed at 50 min, except those related to amino acid metabolism. Genes related to programmed cell death (GO:0012501; GO:0031640; GO:0019835) and phosphate ion transport (GO:0006817) were also upregulated at 50 min. Transcription levels of genes responsible for nickel cation transport (GO:0015675) were downregulated, except for genes encoding phosphate ABC transporter permease subunits PstA and PstC.

Upon linezolid treatment at 5 min, only two enriched GO groups were detected: ‘*de novo*’ IMP biosynthetic process (GO:0006189) with upregulated genes, and maltodextrin transmembrane transport (GO:0042956) with downregulated genes. At 20 min, most genes with increased transcription were also associated with the ‘*de novo*’ IMP biosynthetic process (GO:0006189), with a smaller portion involved in nitrate assimilation (GO:0042128). In contrast, genes involved in the riboflavin biosynthetic process (GO:0009231) were downregulated. At the 30-min time point, two categories with downregulated genes were detected: ‘*de novo*’ IMP biosynthetic process (GO:0006189) and translation (GO:0006412). The GO category associated with cell adhesion (GO:0007155) contained upregulated genes, except for those encoding the metal ABC transporter substrate-binding protein and zinc ABC transporter substrate-binding lipoprotein AdcA. The term related to nickel cation transport (GO:0015675) contained both upregulated and downregulated genes, while genes involved in the small molecule metabolic process (GO:0044281) were exclusively downregulated. The final time point, 50 min, similarly included ‘*de novo*’ IMP biosynthetic process (GO:0006189) and translation (GO:0006412), and also identified the proteolysis category (GO:0006508), mostly with upregulated genes, except for those encoding Abi family protein, signal peptidase II, and zinc metalloproteinase aureolysin.

Under combined treatment with both phage and linezolid, at the 5-min time point, genes associated with pyrimidine nucleotide biosynthesis (GO:0044205; GO:0006207) were upregulated, while nitrate assimilation (GO:0042128), tricarboxylic acid cycle (GO:0006099), and maltodextrin transmembrane transport (GO:0042956) were downregulated. At 20 min, upregulated genes were found in groups related to cytolysis in another organism (GO:0051715) and programmed cell death (GO:0012501; GO:0019835), while only one downregulated group was identified—glycine decarboxylation via the glycine cleavage system (GO:0019464). The 30-min time point showed a similar set of upregulated categories, including cytolysis in another organism (GO:0051715), programmed cell death (GO:0012501; GO:0019835), and viral tail assembly (GO:0098003). Genes with reduced transcription at 30 min were observed in the tricarboxylic acid cycle (GO:0006099) and lactose metabolic process (GO:0005988). The final 50-min time point included only upregulated genes, associated with processes such as cytolysis in another organism (GO:0051715), nitrate assimilation (GO:0042128), and prophage region activation (GO:0046718; GO:0098003).

## 4 Discussion

In this study, we conducted a comprehensive investigation into the combined effect of linezolid and the lytic phage vB_SauM-515A1 against *S. aureus* SA0413Rev. This strain belongs to the highly prevalent clonal complex CC8, one of the most widespread clones both in Russia (Gostev et al., 2017) and globally (Turner et al., 2019), known for causing hospital-acquired infections. The selected phage is a classical representative of the *Herelleviridae* family, considered one of the most promising for therapeutic use (Kornienko et al., 2023).

Our results demonstrate that the combination of phage vB_SauM-515A1 and linezolid against planktonic *S. aureus* SA0413Rev cells led to a synergistic effect. Similar findings of synergistic interactions between antimicrobial agents against *S. aureus* have been previously demonstrated using the checkerboard assay, showing synergy between sub-inhibitory concentrations of linezolid and a phage cocktail that included a member of the *Herelleviridae* family (Prazak et al., 2019). Conversely, an antagonistic effect was observed with the combination of another phage, PYOSa (*Herelleviridae*), and linezolid (Berryhill et al., 2021). Through CFU and PFU counts before and after treatment with antibacterial agents (over 24 h), the authors showed that bacteriostatic antibiotics, including linezolid, inhibited the complete lysis of bacterial cells, regardless of the order of treatment. Moreover, final concentration of phage particles decreased with simultaneous treatment of antibiotics. Notably, in that study, linezolid was used at a super-MIC concentration of 10 μg/mL, likely critically limiting the availability of the bacterial translational machinery for phage propagation. A similar result was observed, where linezolid at the same concentration (10 μg/mL) limited the amplification of a phage mixture that included bacteriophage K (*Herelleviridae*) (Prazak et al., 2021).

In our study, we also observed a negative effect of sub-inhibitory linezolid concentrations on phage replication, reflected by a prolonged latent period (from 20 to 30) and an overall extension of the phage life cycle (from 60 to 100) (Supplementary Figure 1). However, this limitation did not significantly impact the lytic activity of phage vB_SauM-515A1 (Figure 1). In contrast, it was previously demonstrated that sub-inhibitory linezolid concentrations (1 μg/mL) shortened the adsorption time and latent period while increasing progeny release for phage MR-5 (*Herelleviridae*) (Kaur et al., 2012).

In summary, linezolid does affect the phage life cycle, with concentration likely playing a crucial role in the overall effect on planktonic cells. Additionally, the observed differences could be attributed to variations in pre-treatment times with the antibiotic and the specific interactions between the phage and the bacterial strain.

The study of the ability of antimicrobial agents to eliminate biofilm and the bacterial cells within it, as presented here, confirmed the importance of considering the sequence of agent administration—a point highlighted in several studies (Akturk et al., 2023; Dickey and Perrot, 2019; Kumaran et al., 2018; Tkhilaishvili et al., 2018; Wang et al., 2020). In our case, a sequential application led to antagonism, while synergy was observed with simultaneous administration. Synergy from the simultaneous use of phage PYO (*Herelleviridae*) and various antibiotics, including linezolid (2 MIC), against *S. aureus* strain Newman was also reported (Dickey and Perrot, 2019). Notably, in that case, synergy was also observed with sequential administration, aligning with other studies (Kumaran et al., 2018; Wang et al., 2020). These discrepancies could be explained by strain-specific differences in *S. aureus* (Wang et al., 2020). One possible factor is the variation in biofilm formation capacity between strains. The *S. aureus* Newman strain forms weak biofilms (Cue et al., 2015), whereas the *S. aureus* SA0413Rev strain is a moderate biofilm producer, which may influence the resulting effect. Additionally, the choice of phage may play a role, particularly its ability to disrupt biofilm integrity, thereby increasing permeability to antibiotics (Rahman et al., 2011). It appears that phage vB_SauM-515A1, when used alone, is not effective in targeting biofilms, as confirmed by our SEM results.

It is worth noting that, despite the observed synergistic effects in our study, complete elimination of cells from biofilms was not achieved. This finding aligns with previous research and highlights the ongoing challenges in combating biofilm-associated infections (Akturk et al., 2023; Kumaran et al., 2018). A promising approach to address this issue could be the combination of phages and/or antibiotics with agents that degrade the biofilm matrix, such as phage depolymerases. Such a strategy has the potential to enhance treatment efficacy by improving the accessibility of antimicrobial agents to bacterial cells (Knecht et al., 2020; Duarte et al., 2024).

To further investigate the mechanisms underlying the combined action of these antibacterial agents, we performed transcriptional analysis. This approach has been widely applied in phage-bacteria interaction studies, including those focusing on *Herelleviridae* staphylophages (Arroyo-Moreno et al., 2022; Finstrlová et al., 2022; Kuptsov et al., 2022; Kornienko et al., 2023). The transcriptional profile of phage vB_SauM-515A1 used in this study has been previously characterized in detail (Kornienko et al., 2020a), along with the host strain SA515 (ST8) response to phage infection (Kuptsov et al., 2022).

In this study, transcriptional profiles were obtained for bacteria treated individually with linezolid, bacteriophage, or their combination. The presence of shared DEGs across all conditions suggests a nonspecific cellular response to the antimicrobial agents. This response involved a reduction in the transcription of genes associated with the utilization of alternative carbon sources, such as maltodextrin. Changes were also observed in energy metabolism, specifically nitrate assimilation, including the transcription of genes encoding parts of the nitrate reductase complex (*narI*, *narJ*) and nitrite reductase (*nirB*). During the early stages of infection, these genes were downregulated under phage treatment but upregulated with linezolid. In the combined treatment, transcription levels of the nitrate assimilation genes initially decreased and then increased, likely reflecting bacterial attempts to compensate for energy deficits to enhance survival.

In the case of the combined treatment, most DEGs reflected the effect of the phage (Figure 4), indicating a typical productive phage infection. This was characterized by alterations in pyrimidine biosynthesis, virulence-related genes transcription, and prophage region activity, consistent with previous findings (Arroyo-Moreno et al., 2022; Finstrlová et al., 2022; Kuptsov et al., 2022). Specifically, we observed upregulation of leukocidin genes located within prophage regions (region 1: ACIV1F_000348; region 4: ACIV1F_001786; region 5: ACIV1F_002149; region 6: ACIV1F_002288; ACIV1F_002314; ACIV1F_002315) as well as hemolysin genes located outside these regions (ACIV1F_001708; ACIV1F_002746; ACIV1F_002747; ACIV1F_002748).

Differences were also observed in the transcriptional response of *S. aureus* SA0413Rev to phage infection, both with and without the antibiotic, compared to the host strain SA515’s response to the same phage (Kuptsov et al., 2022). In the host strain, transcription of genes associated with nucleotide metabolism was reduced, while in SA0413Rev, as well as in *S. aureus* SH1000 and *S. aureus* Newman infected with phage K (*Herelleviridae*), these genes were upregulated (Finstrlová et al., 2022). Additionally, changes in amino acid metabolism were detected in SA515’s transcriptional response to phage infection, but no significant changes were observed when phage and linezolid were applied together (Kuptsov et al., 2022).

Treatment of *S. aureus* SA0413Rev with phage vB_SauM-515A1, both with and without linezolid, led to increased transcription of the genes encoding a holin-like protein CidA and an antiholin-like proteins LrgA and LrgB. In contrast, phage infection in the host strain only elevated transcription of LrgB (Kuptsov et al., 2022). These proteins play key roles in biofilm formation and maintenance, as they regulate the balance between cell lysis and survival (Ranjit et al., 2011). During cell lysis, extracellular DNA is released, becoming part of the biofilm matrix and thereby strengthening its structure (Mann et al., 2009). This may explain why prior phage treatment restricts linezolid access to biofilm cells, contributing to the observed antagonism during sequential treatment. These variations highlight the strain-specific nature of bacterial responses to antimicrobial agents.

In the combined treatment, two unique enriched categories were identified: glycine decarboxylation via the glycine cleavage system (GCS) and lactose metabolism. Transcription levels of the genes in these categories were reduced, except for the YafY family transcriptional regulator gene. The suppression of these processes can have significant negative effects on the overall cellular metabolism and fitness. The GCS is closely linked to one-carbon metabolism, which is essential for nucleotide and amino acid synthesis. Limiting access to alternative carbon sources, such as lactose, reduces the ability of bacteria to generate energy, leading to slower growth. Thus, the simultaneous action of the phage and linezolid induces a broader metabolic imbalance by restricting the synthesis of key metabolites and energy production.

Analysis of the strain’s transcriptional response to linezolid, in addition to the nonspecific response described above, revealed changes in translation (GO:0006412), specifically upregulation of ribosomal protein genes, consistent with linezolid’s known mechanism of action (Gao et al., 2020). During combined treatment, transcription of genes encoding both large (ACIV1F_000615; ACIV1F_001627; ACIV1F_002546; ACIV1F_002564) and small (ACIV1F_001630; ACIV1F_002560; ACIV1F_002565) ribosomal subunit proteins also changed, though the GO term for translation was not significantly enriched. Additionally, following 30 min of antibiotic exposure, there was increased transcription of genes encoding the MSCRAMMs (microbial surface components recognizing adhesive matrix molecules), which play a crucial role in *S. aureus* biofilm formation and overall virulence by promoting adhesion, colonization, and immune evasion (Foster, 2019).

Here, we reported a comprehensive evaluation of the combined effect of phage vB_SauM-515A1 and linezolid on the *S. aureus* strain SA0413Rev. This combination approach requires careful selection of antibacterial agent conditions (concentration, administration order, phage type, and antibiotic) to avoid potential negative outcomes, such as antagonistic effects. This consideration is important for both planktonic cells and biofilms, though it is particularly applicable to planktonic cells due to the absence of antagonism. Our analysis of the transcriptional response of SA0413Rev revealed significant changes in several essential biological processes. At all time points, the influence of both antibacterial agents was identified, with the phage contributing a larger impact, likely due to its high concentration (10 MOI). It is common practice to use high phage concentrations in studies of bacterial transcriptional responses to phage infection to ensure synchronized infection (Yang et al., 2019; Finstrlová et al., 2022). However, this approach may impose limitations on studying combined treatments. On the other hand, using lower phage concentrations might result in insufficient phage particles to detect a bacterial response. Validation of this approach requires further investigation in future studies.

## Data Availability

The original contributions presented in the study are publicly available in the NCBI repository. This data can be found here: https://www.ncbi.nlm.nih.gov/genbank, accession number CP173176.

## Footnotes

1
https://github.com/rrwick/Filtlong

2
https://github.com/nanoporetech/medaka

3
https://cge.cbs.dtu.dk/services/VirulenceFinder/

4
https://github.com/dbespiatykh/gopiscator

5
https://CRAN.R-project.org/package=ggnewscale

6
https://CRAN.R-project.org/package=ggh4x

7
https://wilkelab.org/gridtext/index.html

8
https://www.statsmodels.org/v0.13.5/index.html
