# Supplementary material for: Response of Staphylococcus aureus to combination of virulent bacteriophage vB_SauM-515A1 and linezolid
Source: Front Microbiol. 2024 Dec 20;15:1519312. doi: 10.3389/fmicb.2024.1519312 (PMC11695419; doi:10.3389/fmicb.2024.1519312)
Supplement: Supplementary file 1 [file Data_Sheet_1.zip › Supplementary Figure 1.pdf]

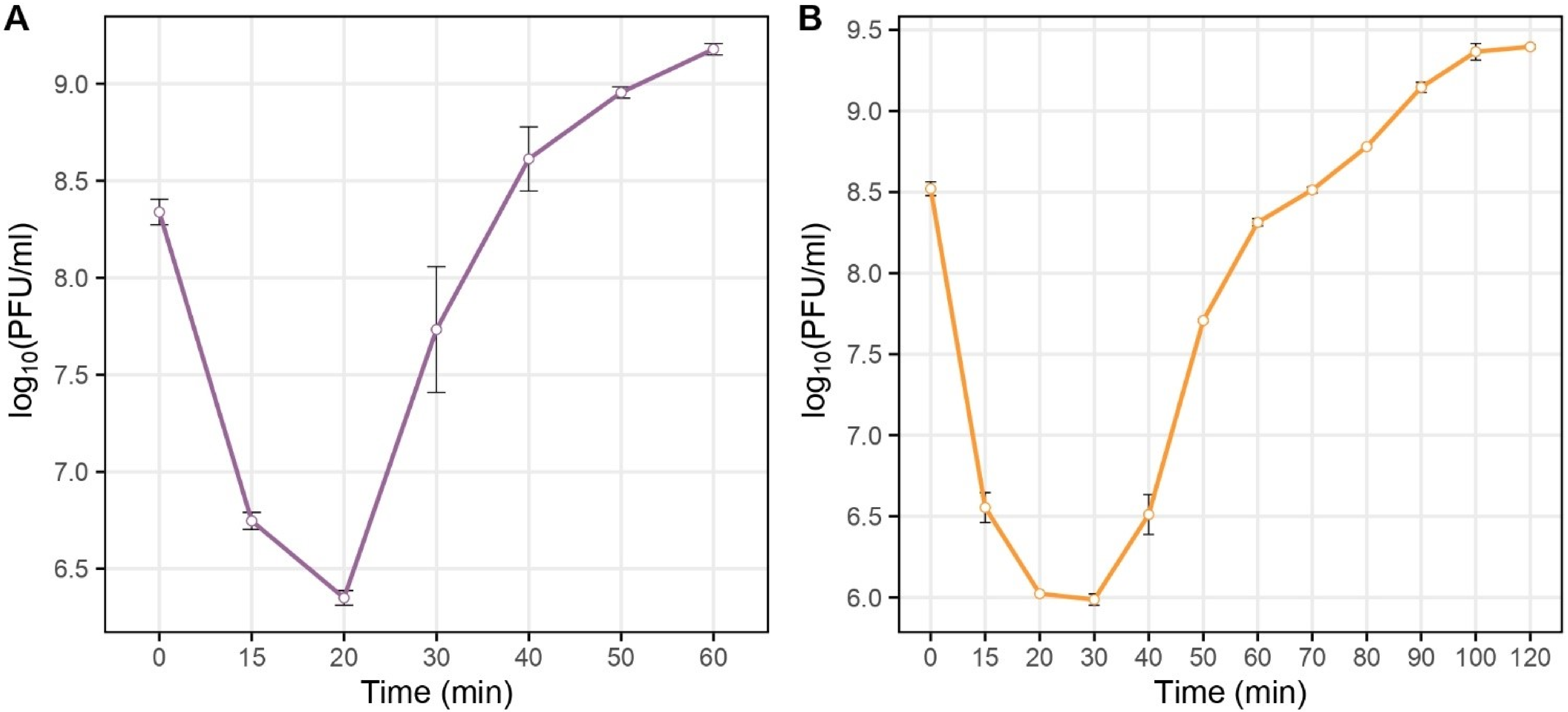

Figure S1. One-step growth curves of bacteriophage vB_SauM-515A1 on *S. aureus* strain SA0413Rev. (A) Without addition of linezolid; (B) with addition of linezolid at a concentration of 1/4 MIC.
