# Supplementary material for: Response of Staphylococcus aureus to combination of virulent bacteriophage vB_SauM-515A1 and linezolid
Source: Front Microbiol. 2024 Dec 20;15:1519312. doi: 10.3389/fmicb.2024.1519312 (PMC11695419; doi:10.3389/fmicb.2024.1519312)
Supplement: Supplementary file 1 [file Data_Sheet_1.zip › Supplementary Text.docx]

Supplementary Text.

The complete genome sequence of *S. aureus* strain SA0413Rev consisted of one circular chromosome (2,993,466 bp; CP173176) with an overall GC content of 32,9% and three circular plasmid sequences of 4,439 (CP173177); 2,366 (CP173179) and 2,908 (CP173178) bp, respectively. The sequences of the genome and the plasmids predicted a total of 3,069 genes, including 2,898 genes that code for proteins, 82 genes for various RNAs (ribosomal/transfer RNA). The presence of prophage regions within the genome sequence was detected using the PHASTEST (Table 1). The genome was characterized by the presence of antibiotic resistance determinants (Table 2) and virulence factors (Table 3) using ResFinder and Virulence Finder, respectively.

**Table 1**. Prophages of *S. aureus* strain SA0413Rev.

| Region | Region length | Completeness | Score | Total Proteins | Region Position | Most Common Phage | Genus | GC % |
| --- | --- | --- | --- | --- | --- | --- | --- | --- |
| 1 | 41.1Kb | intact | 150 | 67 | [355340-396513](https://phastest.ca/submissions/ZZ_f3c513d57f?batch_id=BB_f665795024#region_dna0) | PHAGE_Staphy_P630_NC_048635(24) | *Biseptimavirus* | 33,70 |
| 2 | 64.2Kb | intact | 150 | 74 | [1281275-1345516](https://phastest.ca/submissions/ZZ_f3c513d57f?batch_id=BB_f665795024#region_dna1) | PHAGE_Staphy_phi2958PVL_NC_011344(40) | *Triavirus* | 32,82 |
| 3 | 10Kb | questionable | 70 | 15 | [1556477-1566525](https://phastest.ca/submissions/ZZ_f3c513d57f?batch_id=BB_f665795024#region_dna2) | PHAGE_Staphy_SPbeta_like_NC_029119(3) | n/d | 30,16 |
| 4 | 47.1Kb | intact | 150 | 69 | [1771207-1818325](https://phastest.ca/submissions/ZZ_f3c513d57f?batch_id=BB_f665795024#region_dna3) | PHAGE_Staphy_53_NC_007049(24) | *Dubowvirus* | 33,62 |
| 5 | 67.1Kb | intact | 150 | 68 | [2081264-2148394](https://phastest.ca/submissions/ZZ_f3c513d57f?batch_id=BB_f665795024#region_dna4) | PHAGE_Staphy_53_NC_007049(23) | *Dubowvirus* | 33,36 |
| 6 | 49.7Kb | intact | 150 | 64 | [2199539-2249278](https://phastest.ca/submissions/ZZ_f3c513d57f?batch_id=BB_f665795024#region_dna5) | PHAGE_Staphy_SA345ruMSSAST8_NC_048713(37) | *Biseptimavirus* | 32,59 |

**Table 2**. Determinants of antibiotic resistance of *S. aureus* strain SA0413Rev.

| Antimicrobial | Class | WGS-predicted phenotype | Genetic background |
| --- | --- | --- | --- |
| gentamicin | aminoglycoside | Resistant | aac(6')-aph(2'') (aac(6')-aph(2'')_M13771) |
| tobramycin | aminoglycoside | Resistant | aac(6')-aph(2'') (aac(6')-aph(2'')_M13771) |
| streptomycin | aminoglycoside | Resistant | aac(6')-aph(2'') (aac(6')-aph(2'')_M13771) |
| amikacin | aminoglycoside | Resistant | aac(6')-aph(2'') (aac(6')-aph(2'')_M13771) |
| isepamicin | aminoglycoside | Resistant | aac(6')-aph(2'') (aac(6')-aph(2'')_M13771) |
| dibekacin | aminoglycoside | Resistant | aac(6')-aph(2'') (aac(6')-aph(2'')_M13771) |
| kanamycin | aminoglycoside | Resistant | aac(6')-aph(2'') (aac(6')-aph(2'')_M13771) |
| netilmicin | aminoglycoside | Resistant | aac(6')-aph(2'') (aac(6')-aph(2'')_M13771) |
| fortimicin | aminoglycoside | Resistant | aac(6')-aph(2'') (aac(6')-aph(2'')_M13771) |
| ciprofloxacin | quinolone | Resistant | grlA (p.S80F), gyrA (p.S84L) |
| amoxicillin | beta-lactam | Resistant | mecA (mecA_BX571856) |
| amoxicillin+clavulanic acid | beta-lactam | Resistant | mecA (mecA_BX571856) |
| ampicillin | beta-lactam | Resistant | mecA (mecA_BX571856) |
| ampicillin+clavulanic acid | beta-lactam | Resistant | mecA (mecA_BX571856) |
| cefepime | beta-lactam | Resistant | mecA (mecA_BX571856) |
| cefixime | beta-lactam | Resistant | mecA (mecA_BX571856) |
| cefotaxime | beta-lactam | Resistant | mecA (mecA_BX571856) |
| cefoxitin | beta-lactam | Resistant | mecA (mecA_BX571856) |
| ceftazidime | beta-lactam | Resistant | mecA (mecA_BX571856) |
| ertapenem | beta-lactam | Resistant | mecA (mecA_BX571856) |
| imipenem | beta-lactam | Resistant | mecA (mecA_BX571856) |
| meropenem | beta-lactam | Resistant | mecA (mecA_BX571856) |
| piperacillin | beta-lactam | Resistant | mecA (mecA_BX571856) |
| piperacillin+tazobactam | beta-lactam | Resistant | mecA (mecA_BX571856) |
| lincomycin | lincosamide | Resistant | erm(C) (erm(C)_M13761) |
| clindamycin | lincosamide | Resistant | erm(C) (erm(C)_M13761) |
| erythromycin | macrolide | Resistant | erm(C) (erm(C)_M13761) |
| tetracycline | tetracycline | Resistant | tet(K) (tet(K)_U38656) |
| doxycycline | tetracycline | Resistant | tet(K) (tet(K)_U38656) |
| quinupristin | streptogramin b | Resistant | erm(C) (erm(C)_M13761) |
| pristinamycin ia | streptogramin b | Resistant | erm(C) (erm(C)_M13761) |
| virginiamycin s | streptogramin b | Resistant | erm(C) (erm(C)_M13761) |
| chloramphenicol | amphenicol | Resistant | cat(pC194) (cat(pC194)_NC_002013) |

**Table 3.** Virulence factors of *S. aureus* strain SA0413Rev.

| Virulence factors | Related genes | Gene id |
| --- | --- | --- |
| Adherence | | |
| Autolysin | *atl* | ACIV1F_001886 |
| Cell wall associated fibronectin binding protein | *ebh* | ACIV1F_001413 |
| Clumping factor A | *clfA* | ACIV1F_000887 |
| Elastin binding protein | *ebp* | ACIV1F_001364 |
| Fibrinogen binding protein | *efb* | ACIV1F_001712 |
| Fibronectin binding proteins | *fnbA* | ACIV1F_002830 |
|  | *fnbB* | ACIV1F_002829 |
| Intercellular adhesin | *icaA* | ACIV1F_003012 |
|  | *icaB* | ACIV1F_003014 |
|  | *icaC* | ACIV1F_003015 |
|  | *icaD* | ACIV1F_003013 |
|  | *icaR* | ACIV1F_003011 |
| Ser-Asp rich fibrinogen-binding proteins | *sdrC* | ACIV1F_000636 |
|  | *sdrD* | ACIV1F_000637 |
|  | *sdrE* | ACIV1F_000638 |
| Staphylococcal protein A | *spa* | ACIV1F_000099 |
| Enzyme | | |
| Cysteine protease | *sspB* | ACIV1F_001892 |
|  | *sspC* | ACIV1F_001893 |
| Hyaluronate lyase | *hysA* | ACIV1F_002523 |
| Lipase | *lip* | ACIV1F_003016 |
| Serine V8 protease | *sspA* | ACIV1F_001891 |
| Serine protease | *splA* | ACIV1F_002004 |
|  | *splB* | ACIV1F_002003 |
|  | *splC* | ACIV1F_002002 |
|  | *splD* | ACIV1F_002001 |
|  | *splE* | ACIV1F_002000 |
|  | *splF* | ACIV1F_001999 |
| Staphylocoagulase | *coa* | ACIV1F_000215 |
| Staphylokinase | *sak* | ACIV1F_002252 |
| Thermonuclease | *nuc* | ACIV1F_000891; ACIV1F_001530 |
| Immune evasion | | |
| AdsA | *adsA* | ACIV1F_000025 |
| Capsule | Undetermined | ACIV1F_000141; ACIV1F_000143; ACIV1F_000145; ACIV1F_000146; ACIV1F_000147; ACIV1F_000148; ACIV1F_000149; ACIV1F_000150; ACIV1F_000151; ACIV1F_000152; ACIV1F_000153; ACIV1F_000154; ACIV1F_000155; ACIV1F_000156 |
| SCIN | *scn* | ACIV1F_002250 |
| Sbi | *sbi* | ACIV1F_002745 |
| Secretion system | | |
| Type VII secretion system | *esaA* | ACIV1F_000275 |
|  | *esaB* | ACIV1F_000277 |
|  | *esaD* | ACIV1F_000284 |
|  | *esaE* | ACIV1F_000282 |
|  | *esaG* | ACIV1F_000285; ACIV1F_000289; ACIV1F_000290; ACIV1F_000292; ACIV1F_000295; ACIV1F_000296; ACIV1F_000297; ACIV1F_000298; ACIV1F_000299 |
|  | *essA* | ACIV1F_000276 |
|  | *essB* | ACIV1F_000278 |
|  | *essC* | ACIV1F_000279 |
|  | *esxA* | ACIV1F_000274 |
|  | *esxB* | ACIV1F_000281 |
|  | *esxC* | ACIV1F_000280 |
|  | *esxD* | ACIV1F_000283 |
| Toxin | | |
| Alpha hemolysin | *hly/hla* | ACIV1F_001708 |
| Delta hemolysin | *hld* | ACIV1F_002331 |
| Enterotoxin A | *sea* | ACIV1F_002257 |
| Exotoxin | *set21* | ACIV1F_000471 |
|  | *set30* | ACIV1F_000465 |
|  | *set31* | ACIV1F_000466 |
|  | *set32* | ACIV1F_000467 |
|  | *set33* | ACIV1F_000469 |
|  | *set34* | ACIV1F_000470 |
|  | *set36* | ACIV1F_000472 |
|  | *set37* | ACIV1F_000473 |
|  | *set38* | ACIV1F_000474 |
|  | *set39* | ACIV1F_000475 |
|  | *set40* | ACIV1F_000479 |
| Gamma hemolysin | *hlgA* | ACIV1F_002016; ACIV1F_002746 |
|  | *hlgB* | ACIV1F_002748 |
|  | *hlgC* | ACIV1F_002747 |
| Leukotoxin D | *lukD* | ACIV1F_002015 |
